# P01 IV antimicrobial review…who #CARES?

**DOI:** 10.1093/jacamr/dlaf118.008

**Published:** 2025-07-14

**Authors:** K Atack, S Hackney, L Nguyen, Z Tariq, S Nazir, S Catt, T Wolf, E O’Cofaigh

**Affiliations:** Leeds Teaching Hospitals Trust (LTHT), Leeds, UK; Leeds Teaching Hospitals Trust (LTHT), Leeds, UK; Leeds Teaching Hospitals Trust (LTHT), Leeds, UK; Leeds Teaching Hospitals Trust (LTHT), Leeds, UK; Leeds Teaching Hospitals Trust (LTHT), Leeds, UK; Leeds Teaching Hospitals Trust (LTHT), Leeds, UK; Leeds Teaching Hospitals Trust (LTHT), Leeds, UK; Leeds Teaching Hospitals Trust (LTHT), Leeds, UK

## Abstract

**Background:**

IV antimicrobial review is a marker of antimicrobial stewardship, and national guidance from the UK Health & Security Agency (UKHSA) stipulates trusts should be aware of their performance to drive improvement. LTHT previously counted IV antimicrobial reviews via the clinical review function on the electronic drug chart (eMeds), relying on clinicians to manually complete this, with less than 10% achieved monthly. A pharmacist-led audit in March 2024, showed that 81% of IV antibiotics had a 48 h review documented, however collecting this data was very labour intensive and not repeatable on a monthly basis. The Pharmacy Infection Team worked with the Information & Insight digital team to develop a process where clinicians can write #CARES #decision based on the outcome of their review in the patient’s electronic notes and data is digitally, automatically counted. The CARES acronym is from the national ‘Start Smart then Focus’ guideline and stands for ‘Cease, Amend, Refer, Extend & Switch. #CARES was launched at LTHT in September 2024 trust wide.

**Objectives:**

To improve data collection of antimicrobial reviews to allow for monitoring and supporting AMS performance; to improve the number of reviews completed to improve number of patients switched from IV to oral; and to compare data between the new and previous processes.

**Methods:**

Data is automatically collected monthly and presented on the ‘performance data’ section of the Infection homepage on Leeds Health Pathways, which is available for the entire trust to access. This data was reviewed and compared to the clinical review data.

**Results:**

By January 2025, across the whole trust, 9% of IV antibiotics had #CARES documented in their clinical notes. Specialty and Integrated Medicine had the best performance with 23.97% of IV antibiotics having #CARES documented, followed by Abdominal Medicine & Surgery, with 15.54%. Leeds Childrens Hospital, Head & Neck and Chapel Allerton did not have any #CARES documented in Jan 2025.

**Conclusions:**

The #CARES data, compares with the previous clinical review data, a system that had been in place for several years. There has been good uptake in some areas, yet in other areas, much more work needs to be undertaken to raise awareness and engage clinicians in the process to improve their understanding of the benefits of completing #CARES for patients and the organization. Additional work needs to be done, to review the different decisions made and identify areas of improvement where there are higher levels of IV antimicrobials being continued.

